# Neglected Tropical Diseases as a ‘litmus test’ for Universal Health Coverage? Understanding who is left behind and why in Mass Drug Administration: Lessons from four country contexts

**DOI:** 10.1371/journal.pntd.0007847

**Published:** 2019-11-21

**Authors:** Laura Dean, Kim Ozano, Oluwatosin Adekeye, Ruth Dixon, Ebua Gallus Fung, Margaret Gyapong, Sunday Isiyaku, Karsor Kollie, Vida Kukula, Luret Lar, Eleanor MacPherson, Christine Makia, Estelle Kouokam Magne, Dum-Buo Nnamdi, Theobald Mue Nji, Uduak Ntuen, Akinola Oluwole, Helen Piotrowski, Marlene Siping, Marlene Ntsinda Tchoffo, Louis-Albert Tchuem Tchuenté, Rachael Thomson, Irene Tsey, Samuel Wanji, James Yashiyi, Georgina Zawolo, Sally Theobald

**Affiliations:** 1 Department of International Public Health, Liverpool School of Tropical Medicine, Pembroke Place, Liverpool, United Kingdom; 2 Sightsavers, Nigeria Country Office, Kaduna State, Nigeria; 3 Sightsavers, Research Team, Haywards Heath, United Kingdom; 4 COUNTDOWN, Research Foundation for Tropical Diseases and Environment, Buea, Cameroon; 5 COUNTDOWN, Department of Sociology and Anthropology, Faculty of Social and Management Sciences, University of Buea, Buea, Cameroon; 6 Institute of Health Research, University of Allied Sciences, Ho, Volta Region, Ghana; 7 Neglected Tropical Disease Programme, Ministry of Health, Government of Liberia, Monrovia, Monsterrado, Liberia; 8 Social Science Department, Dodowa Health Research Centre, Ghana Health Services, Dodowa, Ghana; 9 Catholic University of Central Africa, Yaoundé, Cameroon; 10 Neglected Tropical Disease Programme, Federal Ministry of Health, Government of Nigeria, Abuja, Nigeria; 11 Centre for Schistosomiasis and Parasitology, Yaoundé, Cameroon; 12 Department of Parasitology, Liverpool School of Tropical Medicine, Pembroke Place, Liverpool, United Kingdom; 13 Institutional Review Board, Dodowa Health Research Centre, Ghana Health Service, Dodowa, Ghana; 14 COUNTDOWN, Department of Microbiology and Parasitology, Faculty of Science, University of Buea, Buea, Cameroon; 15 University of Liberia Pacific Institute for Research and Evaluation, Monrovia, Monsterrado, Liberia; University of Washington, UNITED STATES

## Abstract

**Introduction:**

Individuals and communities affected by NTDs are often the poorest and most marginalised; ensuring a gender and equity lens is centre stage will be critical for the NTD community to reach elimination goals and inform Universal Health Coverage (UHC). NTDs amenable to preventive chemotherapy have been described as a ‘litmus test’ for UHC due to the high mass drug administration (MDA) coverage rates needed to be effective and their model of community engagement. However, until now highly aggregated coverage data may have masked inequities in availability, accessibility and acceptability of medicines, slowing down the equitable achievement of elimination goals.

**Methods:**

We conducted qualitative programmatic analysis across different country contexts through the novel application of the Tanahashi Coverage Framework enhanced by gendered intersectional theory to interrogate different components of programme coverage: availability, accessibility, acceptability, contact and effective. Drawing on communities and health implementers perspectives (using focus groups, interviews, and participatory methods) from varying levels of the health system, across four African country contexts (Cameroon, Ghana, Liberia and Nigeria), we show who is left behind and provide recommendations for programmes to respond.

**Findings:**

We have unmasked inequities in programme delivery that repeatedly leave vulnerable populations underserved in relation to the prevention and treatment of PC NTDs across all components of coverage explored within the Tanahashi framework. Inequities are influenced by health systems challenges and limitations, due to lack of consideration of gender, power and equity issues. Effective treatment for individuals and communities is shaped by individual identities and the intersecting axes of inequity that converge to shape these positions including gender, age, disability, and geography. Health systems are inherently social and gendered thus they become mediators in managing the impact that social and structural processes have on individual health outcomes.

**Significance:**

To our knowledge this is the only paper which has combined a comprehensive equity framework with intersectional feminist theory, to establish a fuller understanding of who is left behind and why in MDA across countries and contexts. Ensuring the most vulnerable have continued access to future treatment options will contribute to the progressive realisation of UHC, allowing the NTD community to continue to support their vision of being a true ‘litmus test’.

## Author summary

Individuals and communities affected by Neglected Tropical Diseases (NTDs) are often the poorest and most marginalised. Some NTDs (lymphatic filariasis, onchocerciasis, soil transmitted helminths, schistosomiasis and trachoma) have been given specific targets for control and elimination by the year 2020. Reaching these goals is important for the attainment of Universal Health Coverage (UHC) as well as the Sustainable Development Goals and has been described as a litmus test. However, few studies have considered how fair progress toward attainment of these goals has been to date. We used qualitative research methods to explore the equity of progress toward these targets across four countries (Ghana, Cameroon, Liberia and Ghana). We used a framework for assessing health services coverage (The Tanahashi Framework) and combined it with gendered intersectional theory (a theory that helps us think about how individuals position of power and privilege shape their experience) to understand who is left behind and why in NTD programme delivery. We found that health systems challenges and limitations due to lack of consideration of gender and equity issues have left vulnerable populations underserved in relation to the prevention and treatment of PC NTDs. Key steps need to be taken at different health system levels to ensure the most vulnerable have continued access to future treatment options. This will contribute to the attainment of UHC, allowing the NTD community to continue to support their vision of being a true ‘litmus test’.

## Introduction

Globally and nationally efforts to address Neglected Tropical Diseases (NTDs) have had many successes but new approaches and analyses are required to equitably attain defined targets and elimination goals. Individuals and communities affected by NTDs are often the poorest and most marginalised; ensuring a gender and equity lens is centre stage will be critical if the NTD community will reach elimination goals, inform Universal Health Coverage (UHC) and ensure no-one is left behind in contributing toward the 2030 United Nations Sustainable Development Goals (SDGs)[1, 2]. NTDs amenable to Preventive Chemotherapy (PC), through Mass Drug Administration (MDA), (including lymphatic filariasis, onchocerciasis, schistosomiasis, soil transmitted helminths and trachoma) have frequently been the focus of control and elimination targets in relation to NTDs[3]. PC-NTDs have been described as a ‘litmus test’ for UHC due to the high MDA coverage rates needed to be effective and their model of community engagement for access and acceptance of medicines [4, 5]. MDA campaigns globally have demonstrated their ability to reach communities viewed by other health providers as ‘hard to reach’ and so are included as a key equity tracer within the SDGs [4, 6, 7]. However, until now highly aggregated MDA coverage data may have also masked inequities in availability, accessibility and acceptability of medicines, slowing down the equitable achievement of elimination goals and even exacerbating health inequalities [8].

There is growing interest and awareness of the importance of better understanding and addressing inequities in global health including how health systems are structured, the utilisation of health services and in experiences of (ill) health and well-being [9, 10]. This is reflected in the current UHC movement which seeks to establish mechanisms to achieve progressive UHC, through the provision of a package of essential services guided by need and prioritised on reaching the most vulnerable first as opposed to an extension of routine health services [11]. This focus has extended to NTDs with recent analysis of the opportunities posed by gender mainstreaming for NTDs [8]; the development and testing of a toolkit on gender, equity and rights within NTD programming by the WHO [7] and an analysis of quantitative data sets from 16 countries that demonstrated that reporting gender disaggregated data is feasible however understanding gendered barriers remains a priority [12]. A global burden of disease study also highlighted differences in Disability Adjusted Life Years (DALYs) by age and gender [13]. Gender however, as a socially constructed concept, varies through space, contexts and time as individuals construct differing roles and identities shaped by broader political, sociocultural and economic factors [14–16]. Therefore focusing on gender in isolation of other intersecting axes of inequity will not be enough to ensure equitable health care delivery [8, 17]. Furthermore, there is a need to better understand commonalities and differences in experience across diverse and changing contexts such as increased levels of urbanisation, migration and fragility [18–22].

**Fig 1:**
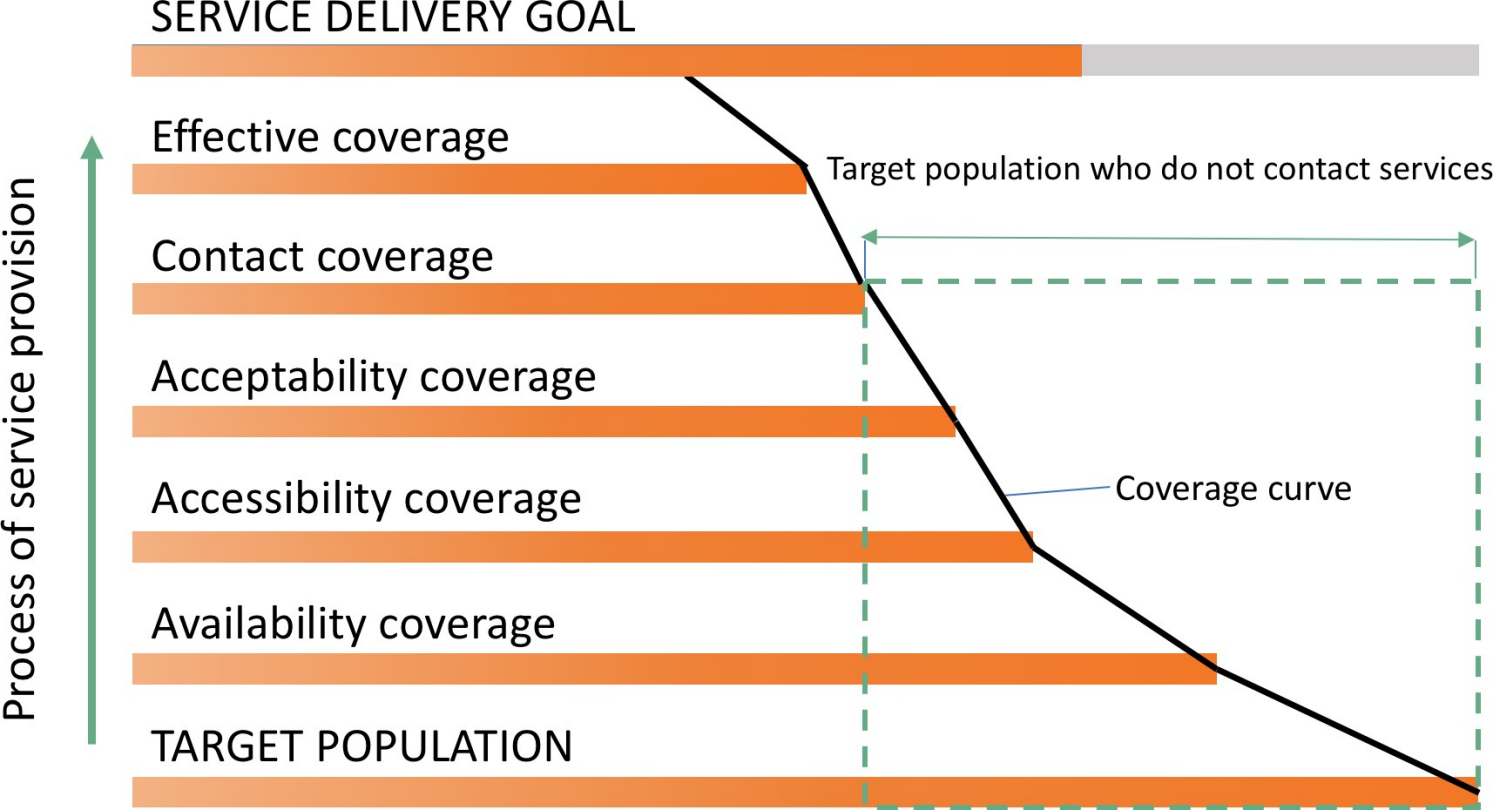
The Tanahashi Framework- an illustration of the links between attainment of service delivery goals and ‘types’ of coverage.

Ongoing equity analysis is clearly not only ethical but essential in monitoring the attainment of UHC including in NTDs. There are many tools and frameworks which aim to promote equity analysis and action. The Tanahashi Framework [23] (see Fig 1), a comprehensive framework which demonstrates how, at each coverage level, various factors within the health system work together to influence who has access to services with the potential to lose people at each stage [23, 24]. Thus, the use of the term coverage in the Tanahashi Framework is slightly different to the common use of the term coverage within PC-NTD programmes. However, the different types of coverage referred to within the Tanahashi framework all contribute to the attainment of ‘drug coverage’ (i.e. the number of persons receiving PC divided by the at-risk population or the eligible population) commonly reported by PC-NTD programmes. Despite being relatively old, the 1978 Tanahashi Framework has gained new momentum in facilitating equity analysis supporting the breakdown of various health systems elements that contribute to quality and effective service delivery whilst also unpacking equity concerns at each stage [24]. The five coverage domains that the model draws upon (availability, accessibility, acceptability, contact, and effective coverage) are appropriate for analysing equitable NTD programme delivery which relies on various components of the health system pulling together to ensure that as many people as possible are in receipt of associated medicines[7, 24] and that the best and most equitable ‘drug coverage’ is attained.

Coverage frameworks alone, do not allow for in-depth exploration of how power shapes underlying context specific social and structural processes that determine who and how certain individuals and populations move up the coverage ladder whilst others are left behind. Gender as one axis of social advantage or disadvantage that can shape marginalisation and impact health is an essential consideration alongside the use of existing coverage frameworks. Gender in isolation however is limiting and we must consider how other individual (e.g. age and dis/ability) and social (e.g. environmental infrastructure) factors interact to shape inequities in service access [20]. Intersectionality seeks to ‘move beyond single or typically favoured categories of analysis (e.g. sex, gender, race and class) to consider simultaneous interactions between different aspects of social identity, as well as the impact of systems and processes of oppression and domination” [25 p3] and is a theory that is increasingly being drawn upon in global health [26, 27]. Intersectionality is not additive, rather it considers how human and social characteristics such as age, gender, sex, ability, disability, race, ethnicity, sexuality etc. interact to shape individual experience at a given point or time. To date, intersectional approaches have not been applied to NTDs; this is a missed opportunity given the importance of thinking critically about programme delivery through time and space, the role of power, how decisions are made and the impacts of these on individuals and communities facing multiple and intersecting marginalities.

The primary aim of this paper is to present qualitative programmatic analysis across different country contexts and apply the Tanahashi Framework intertwined with gendered intersectional theory to interrogate different components of programme coverage. Drawing on perspectives of communities and health implementers from varying levels of the health system across four African country contexts (Cameroon, Ghana, Liberia and Nigeria) our key objective was to understand who is left behind and why within current PC-NTD programming, to allow programmes to think differently about who is not reached and why and to respond. This cross-country study was completed as part of the research programme consortia COUNTDOWN a five-year Department for International Development (DFID) funded project which brings together innovative and multi-disciplinary NTD researchers, policy makers and practitioners to conduct implementation research to produce evidence to contribute to reducing the morbidity, mortality and poverty associated with NTDs [28].

## Methods

NTD control programmes and research on NTDs has primarily focused on programmatic coverage data and other quantitative data sets [29, 30]. Broad data sets can miss unseen or neglected populations and arguably mask specific gender, access and equity issues as they are limited in their ability to provide insights into the norms, processes and relations which shape practice and bring solutions for change [7]. We aimed to specifically understand the experiences, voices and perspectives of communities affected by NTDs, front line providers including Community Drug Distributors (CDDs), teachers and programme managers and hence chose a range of qualitative methods.

### Study settings

The four country contexts were selected as they are the COUNTDOWN partner countries, and each represent different points in the NTD control and elimination journey. Ghana and Cameroon are in latter phases of disease control and elimination for many of the PC NTDs and are moving toward more targeted treatment strategies for ‘hot spot’ areas where there are unexpectedly high levels of ongoing disease transmission. Liberia is comparatively in the earlier stages of disease control and elimination and Nigeria faces the ongoing challenge of how to scale-up the provision of MDA to such a large and diverse population.

### Data collection

Qualitative methods, including key informant interviews, in-depth interviews, focus group discussions and a range of participatory methods (including transect walks, social mapping, seasonal calendars and photovoice) were utilised with purposively selected participants based on the overall COUNTDOWN protocol and adaptations that were made to context. Table 1 gives an overview of which methods were used, with which participants in which contexts. A further breakdown of data and participants can be found in supplementary file one. Variation in methods across contexts enabled triangulation of the findings between type of method, participant group and country context. In all country teams, qualitative data were collected by experienced social scientists in each country who received additional training on trustworthiness in the qualitative research cycle and (where appropriate) the context of NTDs.

**Table 1:** Overview of methods used by context.

Country Context	Study Site(s) within Country	Method Used	Description of Method	Number
Ghana	Nzemah East; Ellembelle; West Gonja; Bole-Bamboi districts	In-depth interviews	Face to face individual interviews were conducted with programme implementers at varying levels of the health system to explore their perspective on the successes and challenges of MDA implementation with focus on identifying what will work in the elimination of LF in Ghana. Individual interviews were also conducted with people who did and did not take MDA within communities to understand reasons for their attitude and practices regarding MDA.	67 interviews 18 interviews
Ghana	Nzemah East; Ellembelle; West Gonja; Bole-Bamboi districts	Focus Group Discussions	Focus group discussions were conducted with different groups of community members based on gender and age to explore general perceptions of MDA and community roles in eliminating LF.	34 focus groups (351 participants)
Ghana	Nzemah East; Ellembelle; West Gonja; Bole-Bamboi districts	Seasonal Calendars	Seasonal Calendars were used to explore the effect of population livelihood activities, seasonality and migration on MDA. Separate groups were conducted with men, women and adolescent males and females.	24 seasonal calendars (264 participants)
Ghana	Shai-Asudoku and Ga South	In-depth Interviews and Focus Group Discussions	Face to face individual and group interviews with health workers, adolescent males and females, adult females and males, teachers and community opinion leaders to explore community understanding of schistosomiasis and STH and the feasibility and acceptability of community wide MDA for Schistosomiasis.	30 interviews 7 focus group discussions (78 participants)
Ghana	Shai-Asudoku	In-depth Interviews, Focus Group Discussions and Vignettes	The participatory vignette method was used to explore community knowledge and experiences on FGS. Participants included in-school and out of school adolescent males and females. Participants for focus group discussions and in-depth interviews were selected on gender and age group basis from the community and the health system. Adolescent females who ever had genital schistosomiasis, community leaders, local medicine sellers, traditional birth attendents, teachers, pupils, health providers, adult males and females were included. To explore community and health provider knowledge and understanding of female genital schistosomiasis and identified potential strategies for giving a voice on the disease to improve the reproductive health needs of women/girls.	34 interviews 11 focus group discussions (99 participants) 6 vignettes (48 participants)
Ghana	Ellembelle	Photovoice	The process involved community drug distributors (CDDs) trained to photograph their everyday work and realities during MDA; then composed written and verbal narratives to accompany the photographs. The purpose of the study was to understand the everyday experiences of the CDDs and the possible impact on their roles in programme implementation. Three (3) females and two (2) males were involved.	5 participants for photo voice
Nigeria	Kaduna and Ogun State	Key Informant Interviews	Key Informant Interviews regarding strengths and weaknesses of ongoing programme implementation with programme implementers at the State and Local government level.	43 interviews
Nigeria	Kaduna and Ogun State (urban and rural LGAs)	Participatory Stakeholder Meetings	A series of stakeholder meetings with frontline implementers (CDDs, Teachers, and Frontline Facility Health Staff) that drew on participatory methods to identify challenges to programme implementation and suggest solutions to overcome challenges.	30 stakeholder meetings (330 participants)
Nigeria	Kaduna and Ogun State (urban and rural LGAs)	Transect walk	Transect walks through the most common route in the community were conducted with influential community members to understand current and potential MDA distribution structures and who is and isn’t reached using these structures.	16 transect walks (77 participants)
Nigeria	Kaduna and Ogun State (Urban and rural LGAs)	Focus Group Discussions	Focus group discussions were held with frontline implementers (CDDs, Teachers, and Frontline Facility Health Staff) to identify the challenges with training and how best to improve training delivery for MDA.	24 focus group discussions (135 participants)
Nigeria	Kaduna and Ogun State (Urban and rural LGAs)	Social Mapping	Social mapping was conducted with community members in groups separated by gender and age. Social mapping involved asking community groups to draw a map of their community identifying key places where key steps in the MDA process (sensitisation, mobilisation, medicine distribution) currently takes place or could take place in the community and who would be or is engaged in these places.	48 social maps (361 participants)
Nigeria	Kaduna and Ogun State (Urban and rural LGAs)	Focus Group Discussions	Focus group discussions were held with community members in groups separated by age and gender to elicit feedback on the best way to communicate messaging and information about the programme.	43 focus group discussions (211 participants)
Nigeria	Kaduna	Focus Group Discussions	Focus group discussions were held with groups of community members and CDDs to explore experiences of programme delivery in relation to accessibility and acceptability of the MDA intervention.	9 focus group discussions (90 participants)
Liberia	National, Maryland and Bong County	Key Informant Interviews	Key informant interviews were conducted with NTD programme implementers at different levels of the health system to explore the realities of MDA implementation from a health systems perspective and focused on what helps and hinders the programme with specific reference to financing, leadership and governance, health workforce and service delivery.	13 interviews
Liberia	Maryland and Bong County	Life and Job Histories	Life histories were used to explore CDDs’ life and career history and elucidate their motivations for the work they do, training they have received, and the ways in which they are supported to fulfil their role. The purpose of these interviews was to understand current levels of job satisfaction and level of engagement with the NTD programme to be able to assess what strategies could be utilised to better support CDDs.	42 life and job histories
Liberia	Maryland and Bong County	Focus Group Discussions including Social Mapping	Focus Group Discussions were conducted with community members to explore general perceptions of Mass Drug Administration (MDA) as well as health communication preferences. FGDs incorporated the use of participatory social mapping to explore community structures (physical and social) that are currently used or could be better used in NTD programme delivery. Separate groups were completed with men, women and youth and influential community members (also separated by gender).	21 focus group discussions (164 participants)
Liberia	Maryland and Bong County	In-depth Interviews with community members	In-depth interviews were conducted with purposively selected community members to understand their knowledge, perceptions and experiences of existing MDA strategies.	40 interviews
Liberia	Maryland County	In-depth interviews with parents of school aged children	In-depth interviews were completed with purposively selected parents of school aged children to understand their knowledge, perceptions and experiences of existing MDA strategies for Schistosomiasis. Mothers and fathers were interviewed separately but as ‘sets’ to try and understand variation in view points and decision making within one household.	19 interviews
Cameroon	South-West Region (Mbonge, Kumba and Bafia Health District)	In-depth interviews	In-depth interviews were conducted with community members to understand their knowledge, experiences and perceptions of onchocerciasis, and experiences of community directed treatment with Ivermectin. The Data captured information from different categories of participants: acceptors and refusers of ivermectin and CDDs, Chiefs of Centres, person living with NTDs, family member of person living with NTDs, and community leaders.	121 interviews
Cameroon	South-West Region (Bafia Health District)	Key Informant Interviews	Key informant interviews were conducted with influential community members (leaders of associations and community leaders) to capture their views on how community directed treatment with ivermectin functions in their communities.	6 interviews
Cameroon	South-West Region (Bafia Health District)	Focus Group Discussions with Community Members	Focus Group Discussions were conducted with community members to capture their experience and perceptions of the community directed treatment with ivermectin	6 focus group discussions (55 participants)
Cameroon	South-West Region (Bafia Health District)	Focus Group Discussions with CDDs	Focus Group Discussions were conducted with CDDs to capture their roles, motivation and perceptions of the community directed treatment with ivermectin	3 focus group discussions (18 participants).
Cameroon	South-West Region (Barombi Kotto and Barombi Mbo Health District) Littoral Region (Edea Health District) Centre Region (Makenene and Yaounde Health District)	Key Informant Interviews In depth interviews	Key informant and in-depth interviews were conducted with key stakeholders at all operational levels of PC-NTD programme delivery (specifically in relation to schistosomiasis, STH and onchocerciasis). The purpose of the interviews was to identify bottlenecks and strengths in existing programme strategies and their implementation to support in designing the way forward for MDA scale up.	163 interviews

### Data analysis

Once collected, data were transcribed verbatim into English and then audio recordings compared to transcripts to check for accuracy in translation by a second reviewer. Data were then analysed using a thematic framework approach [31] within country specific qualitative research teams with support and inputs from specifically trained qualitative researchers within the broader COUNTDOWN consortium. Data were then synthesised, and an analytical account produced to highlight core strengths and weaknesses of the respective programmes with a specific focus on understanding challenges in ensuring equity and access in programme delivery. Where possible initial analyses have been shared and discussed with NTD programme managers leading to some lively interactive sessions. Paper writing workshops, consortium meetings (which brought together researchers from the four country contexts and across the COUNTDOWN partnership) and skype and email exchanges allowed joint analysis across the different domains of the Tanahashi framework and a critical discussion of how gender and other axes of inequity intersect to shape and limit equity of access to MDA. The analytical account is presented below under the core areas of the Tanahashi Framework.

### Ethical considerations

Prior to data collection ethics approval was obtained from the: Liverpool School of Tropical Medicine (15.043); Ghana Health Service ERC and Dodowa Health Research Centre IRB; National Health Research Ethics Committee of Nigeria (approval no NHREC/01/01/2007-19/12/2017); Cameroonian National Ethics Committee for Research on Human Health (approval no. 2016/11/838/CE/CNERSH/SP), and the Division of Health Operations Research within the Cameroonian Ministry of Public Health (approval no. 631–03.17); and University of Liberia Pacific Institute for Research and Evaluation IRB (16-09-009). Written or verbal informed consent was taken from all participants prior to inclusion in the study. Confidentiality has been maintained within data collection, analysis and dissemination (including in the presentation of quotes) through the removal of all identifiable information from transcripts and analysis accounts. All data were collected in private spaces that were acceptable to participants and secure data storage that included separation of consent forms from other data was used. Where the job role of specific health cadres may have made them identifiable due to the minimal numbers or men/women in specific cadres at specific levels of the health system, we took extra care to discuss the way the data were presented with these participants and chose not to report sex, age or specific job role where necessary.

## Results

### Context and commonality: Donor dependency, privilege and oppression, fragility and programme legacy

From an intersectional perspective, it is important to position inequities within geographic contexts and to better understand the political, historical and institutional (health systems and programmatic) trajectories that shape current practice. From our analyses there were many similarities which emerged across all country contexts. One key similarity is the aid dependency and vertical funding streams for different NTDs that impact on programme functioning by limiting both flexibility and autonomy of national NTD programmes.

‘*The donor tells us first*, *‘here are my priorities*, *here is the amount that I can give you…Now*, *you take his priorities as you go into activities* … *there may be [other] activities that are even relevant*, *but because there are no resources*, *we will not finance’… (NTD implementing partner official*, *male*, *IDI*, *Cameroon)*

Disjointed funding streams and a lack of co-ordination between funders and implementing partners exacerbates inequities; for example, cross-border gaps in distribution can result in some communities being missed during MDA campaigns. Illustrative quotes are provided in the subsequent sections to further support this.

Another commonality is that each country context has the challenges of hidden, hard to reach, vulnerable and marginalised individuals and populations (geographically, socially and politically) who face extreme poverty and in some cases conflict and fragility. The levels of fragility shapes context in multiple ways. Liberia’s historic fragility based on conflict and the Ebola epidemic has brought multiple challenges (and opportunities) for the NTD programme. Pockets of recent and ongoing conflict are also occurring in both Cameroon (in the South West Anglophone areas) and Nigeria (mainly in the North and the Niger Delta) and have wide reaching impact on the programme and communities.

Programme legacy matters both from a programmatic perspective and from the views and memories of communities. In Cameroon, where onchocerciasis co-exists with Loa Loa, previous Ivermectin distribution resulted in severe adverse events including some deaths which has presented long term challenges for programmes and community acceptance of other PC NTD drug acceptance. In Nigeria, Cameroon, and Ghana there is a long and sustained history of programme delivery which has obtained many successes. However, through time in all contexts, the initial onus on community directed approaches and flexibility in response has arguably lessened due to both donor dependency/inflexibility and the bureaucratisation/normalisation of approaches.

### Availability coverage

Availability coverage is concerned with ensuring that all services are available to those who require it within a given population, this requires adequate staffing and resources so that the maximum capacity of the service is not limited[23]. Across all contexts, availability coverage was negatively impacted due to staff limitations, medicine shortages and delays in the supply chain leading to equity challenges with some populations being untreated. Over time, updating the community census prior to MDA distribution has ceased in some areas resulting in inaccurate drug requisition requests based on outdated population estimates. Coupled with poor supply chain management, particularly within Nigeria and Liberia, this frequently resulted in localised drug shortages and therefore specific populations being untreated, in some cases decided by political affiliations or relationship with the distributer. Those untreated included people perceived as ‘outsiders’ to communities e.g. migrant communities, and those from different ethnic or religious backgrounds to the majority in specific settlements (for example Nigerian communities in Cameroon) or simply those most remote and furthest from the health facility who got treated last.

In relation to school-based distributions, where schools were large or when the programme did not engage with particular schools (for example Islamia/ Almajiri schools in Nigeria, and private schools in Nigeria and Cameroon) children often went untreated. Out of school children were a population that are particularly hard to reach across all contexts. In Liberia, where more children are out of school than in school, this had led to an adaption of distribution mechanisms to community-based processes to reach coverage targets. In Cameroon, some schools chose to encourage out of school children to come and collect medicines on distribution days but found this still left out many children, stressing the importance of community-based distribution. In Cameroon and Nigeria, teachers expressed a need for stronger support from and links with community drug distributers and the health system to increase parental trust of medicines distributed by teachers and to handle side effects.

*‘…the main bottleneck is private schools. Very few private schools take part in deworming campaigns…when the child is not in school, it becomes complicated for us*. *It’s true that we insist if a child doesn’t attend classes in a school and presents himself on the day of distribution, he should not be denied treatment. But still, it is hard to imagine a child coming to a school which he doesn’t belong to, especially in our schools that have fences.’ (Regional Education official, male, KII, Cameroon)*

*‘Some children came from the community asking for the medicine, but we didn’t have much for them only few of them got because we were instructed to focus on the school enrolled children’ (Teachers, FGD, Kaduna State, Nigeria)*.

*Likewise, in southwest Cameroon schools (primary and secondary) in 20 intervention communities have been closed for over three years due to conflict, so programmes have responded by undertaking community-based distribution which has increased availability. Where drug supply did not meet demand, this often-compromised trust between health and education systems and the communities they were serving. In many cases, CDDs, teachers and health facility staff would do all they could to obtain medicines to reach those currently missed by using their own monies to travel large distances to replenish medicine stocks and follow up those who were previously untreated, this could be impoverishing for implementers. In Ghana, localised drug shortages were uncommon, however staff shortages in some areas hindered some populations (particularly those living in small settlements far from each other) from receiving medicines*.

*‘Look we have so much drugs with us here, this is even from the last two distribution cycle. The problem is that we do not have enough CDDs to distribute these drugs. We can only use few CDDs because the program says there is no money. That is why some people are complaining of not receiving them’ (Subdistrict health staff, Ghana)*.

*‘I find difficulty in that money does not flow for me, that is, when I want to go and get the Mectizan I stress a lot …The little money that I would have used to eat I use it instead to pay the transportation to and fro when I want to go and take Mectizan and also when I want to return it**.’** (CDD*, *Male, IDI, Cameroon)*

In Cameroon and Liberia, there were reports from community members and CDDs that historically, medicines were sometimes kept for selling by various actors within the health system, including CDDs and frontline health facility staff (e.g. chief of post or pharmacists).

*‘ …at times, they used to sell it [Ivermectin]. Like the pharmacist at the health centre, they use to sell it, five hundred frs a tablet, I know that it is the left over from the one you people have been giving. Even the chief of post, the one who was there like that, he used to give her to sell (CDD, Male*, *IDI, Cameroon)*

### Accessibility coverage

Accessibility coverage is concerned with ensuring that services are within reasonable reach of target populations[23]. We identified that the duration, seasonality and method of distribution, when not adequately considered have the potential to negatively impact equity in access to MDA.

#### Duration of distribution

Timing of both awareness activities and medicine distribution was a key factor in shaping equity in programme access. Delays in the supply chain, coupled with stringent reporting structures and timelines from multiple donors, frequently condensed distribution periods across all contexts. Delays in the supply chain also hindered effective sensitisation campaigns. Consequently, community members either did not know about the programme or refused to engage due to lack of knowledge and understanding, thus impacting accessibility coverage. When poor sensitisation had taken place, community members were unable to plan to adjust their routines to gain access, or when the time of day that distribution took place clashed with livelihood or religious activities, specific pockets of the population were missed, particularly when no “mop-up” took place, again due to lack of medicine availability or condensed delivery timelines. Across many contexts, it was frequently men who were absent from communities during delivery due to livelihood activities such as farming, or fishing being concentrated outside of settlement areas (e.g. men in Ghana were frequently absent if engaged in small scale mining or fishing activities). Men often reported that if they had known about the distribution taking place, they would have adjusted their routine; this raises an important consideration about the flexibility of current MDA approaches to ensure health interventions do not negatively impact community members.

*‘There was no time to mobilize the people; to tell them that we will be on the field on Sunday…if we were given at least …even the distribution, of 5 days, it was small…So the five days we were given for the distribution, if we could get eight days, we could work a lot…’ (CDD, Male, IDI*, *Cameroon)*

*‘I am not permanently residing in the town, I most often spend my time in the village, so I can come when necessary…I have not experienced people coming here to talk about river blindness, what cause it, I have not experienced it. I have not seen people coming here to talk about elephantiasis and the causes agent of elephantiasis …’ (Community Member, Female, IDI, Liberia)*.

*‘There's an area in my neighbourhood—I can't lie to you- At [details on location] where I did not enter. Because the time was too short. That's why we're really asking for time for distributions…’ (CDD, Male, IDI, Cameroon)*.

Parents in Liberia and Cameroon described that their children who travelled out of the community with them while they completed livelihood activities also missed distribution. Parents in Ghana complained that their children away in boarding schools were always left out of the exercise and there were no plans by the program to reach them when they finally come home on vacation. In Liberia and Cameroon, some men suggested that completing awareness and distribution over the weekend would mean more people would likely be present. However, in Nigeria, caution was described in completing house to house distribution on Sundays as most Christians would be absent from their homes. Nevertheless, across Cameroon, Liberia and Nigeria religious structures were identified as a key structure through which information or awareness messages could be shared to reach large numbers of the population.

*‘But mainly when it is Sunday everyone will be in the town. So, when you make that announcement Saturday evening, then Sunday the whole place will be park. Sunday is a resting day, and nobody will be able to go on the farm’ (Community Member, IDI, Male, Liberia)*.

*‘For most of them who are Christians, you will be lucky to meet them on Sundays, on the other hand you will be lucky to meet the Muslims on Fridays since they will not go far away from the vicinity of their mosque’ (CDDs, FGD, Nigeria)*.

*‘we have days of working if you want to see a lot of people. We have Sundays where we can announce the distribution program in the church and everybody can hear easily’ (CDD, IDI, Female, Cameroon)*.

Over ten years of MDA in Ghana and Nigeria, the level of engagement and commitment to information and sensitization activities at varying levels of the health system has drastically reduced due to apathy and the assumption that community members are already well informed. Informants described that despite a long legacy of implementation, the programme does not consider that populations have changed and increased with younger community members and inward-migration most of whom do not know the purpose of the MDA and what the drugs are meant for.

*‘Madam, I moved here with my parents three years ago and no one ever told us what the drugs are for and why we should take them. They just come around and write our names and ask us to take the drugs for some disease. I have never allowed them to take it because we are not sure where it comes from.’ (Community Member, Male, FGD, Ghana)*.

#### Seasonality of distribution, programme logistics and hard to reach areas

Seasonality of distribution also shaped programme equity. In Nigeria, Cameroon and Ghana, distribution during the rainy season leads to inability to reach farming populations as well as placing additional burden on CDDs due to negative impacts on their livelihood activities. In Liberia and Ghana during these periods, poor roads and pathways meant that specific communities were cut off and completely inaccessible to CDDs and therefore whole areas remained untreated. In addition, lack of equipment such as protective clothing (rain coats and boots) in Liberia and Ghana compromises the safety and security of the CDDs and makes it very difficult for them to manoeuvre to the difficult forest areas to reach most farming populations.

*‘It was done just as in the past, the only difference being that this year we have gone far into the raining season before it was distributed. Consequently, the men have already gone off to their farms and could not access the medicine. Last year the distribution was done earlier as such everyone, male and female alike could access the medicine. But this year it was done deep into the farming season, so it means more men did not access the medicine due to farm work’ (Older community member*, *male, FGD, Nigeria)*

*‘the programme does not consider our health and safety. We do not get even coats or boots to protect us from the heavy rains and harmful things like snakes and scorpions. We risk our lives for nothing’ (CDD, Female, IDI, Ghana)*.

In Cameroon and Nigeria, distribution campaigns that took place during religious fasting periods also presented a challenge for CDDs. CDDs did not describe these periods as impacting accessibility of MDA, however, it presents challenges for programmes in being able to accurately record coverage data as directly observing treatment is not possible.

*‘… there is also a problem because there is this moment of fasting which Muslims observe, and at that time they are still fasting…what can I do? I just have to give them the medicines, so that when that time reaches [fasting ends] they can then drink it [CDD, Male, IDI, Cameroon]*.*’*

#### Distribution method

The distribution method used was also of key importance in shaping access to medicines. Participatory community mapping methods utilised across Liberia, Nigeria and Ghana were a particularly useful easy to use tool in identifying key distribution methods. Across all contexts, to ensure maximum inclusion of all community members during MDA both house to house and fixed-point distribution methods were called for by community members. In Liberia, house to house methods tended to be preferred in some rural areas to allow CDDs to identify those who were reluctant to take medicines and to ensure appropriate spread of awareness messaging, in Nigeria and Cameroon it was also indicated to minimise cost and time for community members; however, in other rural areas fixed point distribution was preferred as group distribution was thought to have the potential to increase medicine acceptability. This was particularly true for women who felt more comfortable taking medicines in the presence of their friends. Preferences for fixed point distribution locations varied across contexts based on geography (urban/rural) as well as religion, and gender, although clinics, existing community meeting points, religious structures and market places were overall, highly favoured. In Cameroon and Nigeria, the Chiefs house was a point for distribution which worked well where chiefs were popular but could affect access if they were not liked.

*…some people since they were distributing the drugs in the chief’s palace, they were those who did not like him*, *who were not on his side, so they never went there (CDD, Male, IDI, Cameroon)*

Accessibility of people living with disability in Nigeria and Liberia was also noted by several participants who suggested that house to house methods may be better for individuals living with disability, particularly those with physical impairment who may face mobility restrictions. In one urban context in Nigeria, some people with disability described that they would prefer to be treated at spots where they go to beg.

*‘‘it is good that the health people always come to our homes with the medicines, but we want them to set up at the school or market so that anyone who is not at home can get it there” (Community Member, Male, FGD*, *Ghana)*

*‘it is good because other person in the house can’t walk to the place where they schedule their medicine, so house to house is good’ (Community member, Male, IDI, Liberia)*.

*‘Sister! it does not cost me anything…Mectizan meets me right inside my kitchen and even inside my bed room without any expenditure.’ (Community member, Female*, *FGD, Cameroon)*

### Acceptability coverage

There were several programmatic factors that shaped acceptability of medicines and consequential equity in attainment of coverage goals. For example, across all contexts, historic memories of positive programme experiences and perceived benefit of curing sickness (e.g. expelling worms) were identified as key motivating factors for individuals and communities to swallow medicines. The role of influential community members in shaping the decision to accept or reject medicines was also particularly prominent based on gender and generation. For example, in parts of Nigeria and Cameroon fear within communities that medicines were a form of family planning often led influential community members to challenge the medicine provision resulting in women, girls and school-aged children rejecting medicines. In Liberia, women also described swallowing the medicines because of community leader instruction and a fear of disrespecting authority.

*‘…I was refusing to drink Mectizan because they said it aborts pregnancies, I feared drinking the Mectizan…’(Community member, female, IDI*, *Cameroon)’*

*‘I’ve got plenty sickness in me, so I just took it maybe it will help you …I’ve been taking it from the time they been bringing it …But the past time, the first time never used to. But when I started taking the drugs this few time even when I reading in the night I can sometime see clear’ (Community member, IDI, Female, Liberia)*.

Where community members, or in some parts of Cameroon where the majority of communities refused to swallow medicines, the main reason was due to observed or experienced side effects during previous MDA rounds. In some areas of Cameroon where Loa loa is co-endemic with onchocerciasis, historically, severe adverse events were experienced from taking Ivermectin and so many members refuse MDA because of fear of ‘provoking disease’ or fear of death.

*‘I have not been taking because I am afraid because some people say when they take, they get swollen and others die. So that has made me never to take Mectizan, and I have never taken Mectizan.’ (Community member*, *Male, IDI, Cameroon)*

Side effects experienced were usually described as itching, swelling or vomiting. Backlash from communities because of side effects to CDDs and teachers who were inadequately supported and supervised by the health system was challenging and shaped their willingness and ability to deliver the programme. In Nigeria, in extreme cases demand for teachers to pay for medical care needs attributed to MDA instigated significant out of pocket spending in a context of poverty. Strengthening education, awareness and referral around side effects for health workers and communities would likely increase programme acceptance in these areas. In Ghana, community members who had experienced side effects complained that when they report to the health facility with the symptoms, they are not attended to in time and they also must pay for necessary treatments to manage side effects.

*‘It is my policy and the reason why I was refusing to take it…Because the first one I took it make me to feel bad so I look at it I say I scare to take it again because when I take it just like to say my sickness is coming up …The medicines made me to be sick almost one week, when I took it my something [scrotum] and my foot swollen and I can go in the latrine fast. . . .No I took it that’s what I saying I took it but la [it was] the second time that I didn’t took [take] it …It is my policy and the reason why I was refusing to take it’ (Community member, IDI Male, Liberia)*.

*‘how can you come and give us drugs and when the drugs is causing us problems we don’t know where to go or who to contact. When we go to the hospitals we are charged for medicines again to treat us. I will never take this medicine again even if it will give me new life’ (Community member, Male FGD, Ghana)*.

Economic context interacted with medicine side effects to shape acceptance. In the poorest communities, where children were often sent to school without breakfast, or communities survived on one meal per day, experience of side effects was exacerbated due to children and community members having not eaten prior to distribution. In Nigeria, Cameroon and Liberia, provision of food prior to MDA, sometimes through collaboration with national feeding programmes or school-based feeding programmes had encouraged the acceptance of medicines in these communities, meaning the most impoverished individuals were less likely to be lost to the programme at this stage. In Ghana, community members requested for food to be given to them before the medicine. Some people recommended that the CDDs should carry some biscuits and drinks so that vulnerable groups such as children and the elderly can have something to eat before the medicine is given especially in very impoverished communities. In Nigeria, some CDDs carried water with them (purchased themselves) to encourage community members to accept the medicines. Directly observed treatment became of critical importance when food or water was also distributed with medicines, otherwise there were reports of some community members accepting but not swallowing medicines in order to receive food and water.

*‘Children came to take the medicine because of the school feeding program knowing fully well they will get to eat before or after taking the medicine, but [a] few their parent forbid them from taking the medicine, I witnessed some children after eating the food and giving the medicine, they kept it in their mouth and spit it out later on but we recorded a great success here’ (Teacher, Nigeria)*.

*‘the health sector should provide food for us before we are given the drugs. The CDDs can carry some biscuits and malt to give to the children and old people so that they can be strong to take the medicine’ (Community member, Male IDI, Ghana)*.

Fear of side effects creating additional economic pressures was found in Cameroon. Men feared that side effects would limit livelihood activities and some community members feared side effects would lead to healthcare costs that families could not afford.

Delivery mechanisms also interacted with traditional belief systems to shape programme acceptance, for example, in some parts of Liberia, and in Islamic areas in Nigeria, community members, particularly women, described a preference for not being measured using a stick and suggested that a different way of measurement would increase medicine acceptance as sticks are also used to measure corpses. Finally, historic experiences that led to a breakdown in trust between health systems and communities influenced medicine acceptance. For example, in Liberia, during the Ebola outbreak, community members described being told not to take medicines from anyone, so they refused these medicines due to a lack of additional awareness activities. In very few cases, some participants also described a perception that the medicines were bringing Ebola. However, some mothers and fathers described an increased likelihood to accept medicines in future MDA rounds due to the follow up visits CDDs made to their children during this distribution period to alleviate these fears.

*‘Well this world we living in everybody want good thing about themselves, even though this time we are skeptical in taking the tablet because the Ebola that broke out people said that people were doing bad things about it that even if you are healthy as soon as you take the tablet then you become weak (Community member, Female, IDI, Liberia)*.

### Contact coverage

Contact coverage is determined by the number of people who have contacted the service, that is those people who come into direct contact with the service provider[23]. Contact coverage between those delivering the service and those who we hope to receive it is essential, however, community power relations are of critical importance in determining how this interaction takes place, thus impacting intervention equity. CDDs are usually the main distributor during MDA. Across all communities and contexts, the way in which CDDs were selected was of importance in shaping interactions with the distribution programme. Many communities wanted to be involved in selection of who it was who would distribute the medicines to them and prioritised factors such as belonging to the community, having some prior knowledge of health or drug distribution methods and having links with the local clinic.

*‘somebody must be attached from the health facility before that person can be given the chance to carry out medication around…’ (Community member, female, IDI, Liberia)*.

Across all contexts, more CDDs were described as necessary to reach the whole population, but funding shortages and limitations (as described in the context and commonality section) had reduced the number of CDDs that the programme was able to engage.

*‘When we started MDA, we used to have one or two CDDs per community depending on the size. These days there is one CDD for over ten communities. How can they reach everywhere? It is not possible…’ (sub-district health officer*, *IDI, Ghana)*

CDD selection processes are frequently linked to gender norms, power and patriarchy within communities and households and needs to be negotiated carefully in programme design and delivery. Across multiple contexts, it was identified that the gender of CDDs shaped interactions at household level and may lead to some members of the household, particularly women, being untreated and invisible within programme reporting structures. For example, in parts of Nigeria, where male CDDs cannot enter the household to distribute medicines to females without the household head being present, some females were left untreated. This also presents challenges in ensuring accurate census estimates (when the census occurs) i.e. due to the recording process CDDs undertake, it is possible that if they don’t enter the household, members of the household could become ‘missing’ within data sets recorded in the CDD register.

*‘it means that the men cannot enter the houses even though they are members of the community and were selected by the community most men would not want another man to see their women. This means that if the head of the household is not at home, those people won’t get medicines…’ (LGA Health Staff, IDI, Nigeria)*.

Patriarchal decision-making structures across many contexts, also shaped autonomy in decision making for many community members as described in the acceptability section above. In addition, in northern Nigeria, CDDs also reported that in cases where it was a household head who has not seen benefits to the medicine, they sometimes, in addition to not taking the drugs themselves, prevented other household members from taking them. This was also experienced in Cameroon where household heads did not trust distribution campaigns as they were linked to the government. In this way, the lack of benefits or trust by one person would have a disproportionate impact on coverage.

*‘I have an issue with a particular household. I was told that for the past two years, they have refused to accept the drugs. The head of the family says he has not seen any benefit for taking the drug and in turn has stopped the rest of his household from taking the drugs’ (CDD, FGD, Nigeria)*.

Contact coverage for CDDs was also described as compromised when trying to reach specific population groups including nomadic or transient groups and those living in security compromised areas. Strategies to ensure nomadic and transient groups were identified as: ensuring engagement of health workers from within those areas; recruitment of CDDs from within these populations to encourage their support in programme design and delivery; as well as the lengthening of distribution periods so that nomadic or transient groups may receive treatment when they meet more ‘static’ populations.

*‘I have been now for 15 years and the only people who posed a challenge were the Fulani people who move about the country so when they come back they would have increased, or another separate group will come back. This has made it difficult in time past, so some people will not get the drugs. Now however we have CDDs among them who work with those of us who don’t move around like they do’ (CDD, FGD, Nigeria)*.

In Nigeria, in urban areas residents came out to take the medicines having seen that it was community health extension workers in uniform that were completing the distribution. In some cases, this encouraged those who had refused the medicines previously to swallow the tablets as health workers were trusted to have professional training to carry out health interventions. For instance, some residents felt that they ‘*have adequate knowledge about what they are doing*, *and it was not that they gave out the medicine to just anybody’*. This contributed to improved treatment coverage in those places as parents also allowed their eligible children to take the medicines.

### Effective coverage

Effective coverage is achieved when all those who are eligible to receive the service have had equitable opportunity to obtain treatment. In treating persons living with disabilities, across most contexts, CDDs and programme implementers described trying not to discriminate in determining who they delivered the medicines to and followed guidelines closely. However, they identified that this was a challenge when it came to the treatment of people living with disability (e.g. epilepsy or people who were unable to stand). These scenarios were not covered in their training or there were reports of inconsistent advice or guidelines given by different trainers.

*‘For the physically disabled, they are told to stand on crutches and measurements made then we give the appropriate drug sometimes, at other times we just don’t give them’ (State Health Staff, IDI, Nigeria)*.

Additionally, health workers or CDDs faced difficulties establishing pregnancy status of women, either because enquiry about pregnancy sometimes offended husbands or where women were not aware they were pregnant. There was concern that if pregnant women were not successfully excluded from treatment they miscarry because of the drugs. This resulted in some of these individuals being included and some excluded depending on the circumstance and on a case by case basis. For pregnant women not included in distribution or for those who are sick based on medical grounds, across all contexts, no mechanism for follow up of these populations following MDA campaign periods was identified. Some women who are pregnant or lactating for multiple distribution rounds and those who are chronically sick who in some contexts are perceived as ‘ill’ (for example people living with HIV/AIDS) may, therefore, be constantly missed and thus more vulnerable to ongoing infection or increased morbidity. There is a need to think through innovative strategies to reach these populations outside of routine campaign periods.

Policy decisions such as school-based treatments, can also render coverage ineffective for specific populations who need medicines but are not considered within programme design, as described in the accessibility section above.

## Discussion

### The importance of intersectional gender analysis in evaluating coverage frameworks on the pathway to UHC

Coverage frameworks that allow for consideration of all health systems factors and recognise the limitations of service provision are clearly important. However, when used in isolation they can mask underlying power dynamics that result in inequitable intervention outcomes. There have been few papers to date that use in-depth qualitative research across multiple countries that explore the equity of MDA. To our knowledge this is the only paper which has combined a comprehensive equity framework (the Tanahashi Framework) with intersectional feminist theory, to establish a fuller understanding of who is left behind and why in MDA in different contexts. The diversity of contexts strengthens the rigour of our analysis and enables critical perspectives of communities and health systems actors from different country contexts. We were also able to compare differences and similarities across and within country contexts that add value to the complexities of MDA delivery uncovered through an intersectional analysis, including adaptations required based on geography (urban and rural), religion (Islamic and Christian), post-conflict and fragile settings. Whilst presenting a nuanced account of the depth and detail from every context was not always possible, throughout our analysis we have drawn on the rich contextual understandings of our authorship team and more nuanced accounts of findings can be found in associated published [32] and forthcoming briefs and articles [33–38]. In interpreting the findings presented here, it should be recognised that most of the study team occupy positions as ‘outsiders’ within study communities as may be perceived as occupying relative positions of power in comparison to research participants due to their social position. Although this is true within many research studies, we took several steps to minimise power imbalances. Throughout the data collection process everything was done to build rapport and trust with participants, particularly those perceived to be the most ‘vulnerable’, however, it is still likely that some participants did not feel able to disclose all parts of their experience. Researcher positionality may have also shaped interpretation of participant responses. Participatory dissemination activities with stakeholders at varying levels of the health system were completed to triangulate data interpretation and encourage additional sharing of experience, however this may not have fully mitigated this challenge.

**Fig 2:**
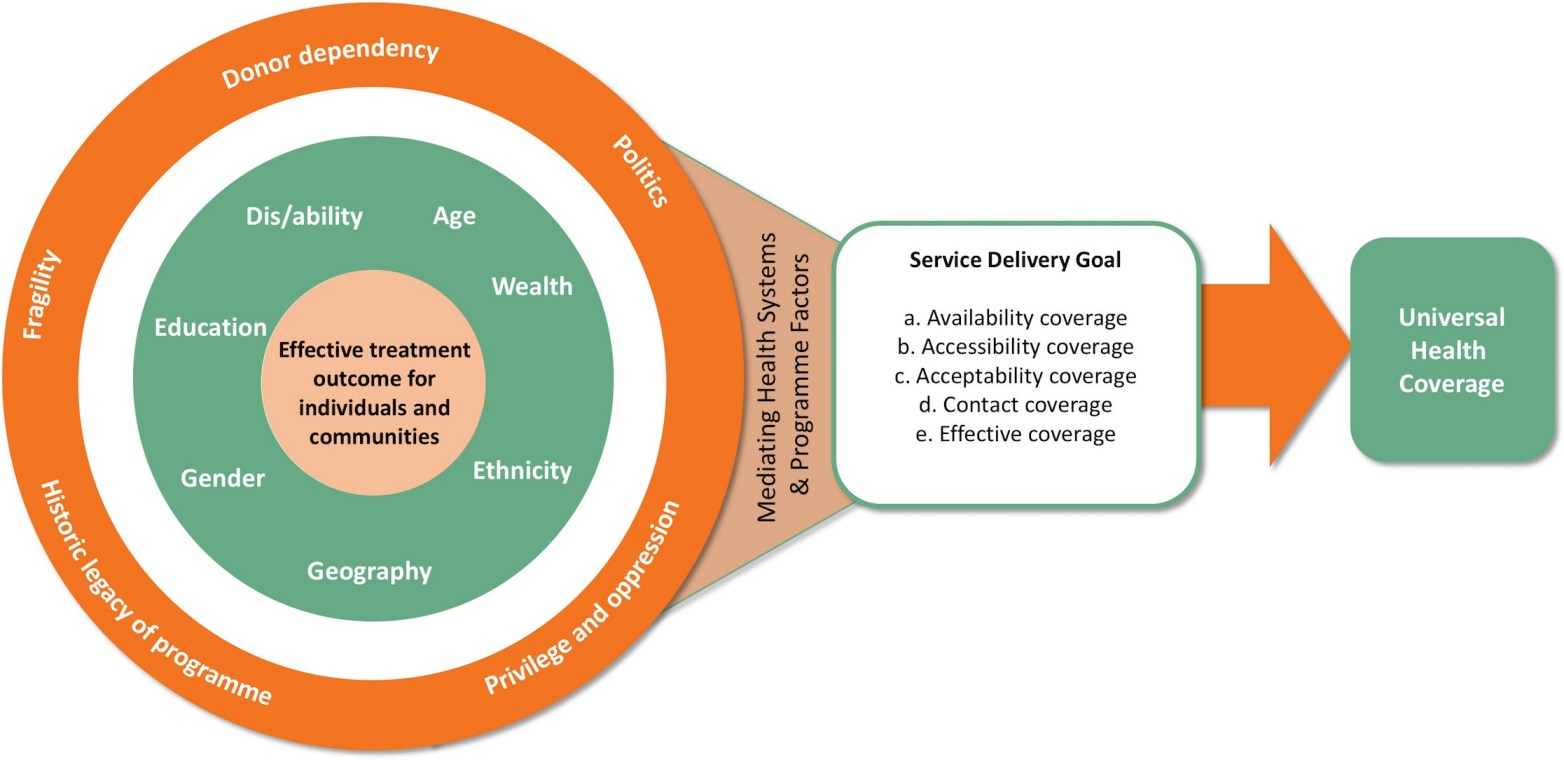
Adapting Simpson’s[39] intersectionality wheel to show how social and contextual factors interact and are mediated by health systems factors to shape equity of NTD programme delivery.

Our findings show that who is missed in MDA is often determined by underlying power dynamics which shape programme delivery structures and intersecting axes of inequity which may vary in different contexts. Acting to address these power imbalances in the delivery of MDA is essential to ensure equitable attainment of service delivery goals and to contribute toward the attainment of UHC. We present the convergence of these factors within Fig 2.

The outer circle of our adapted Simpson’s intersectionality wheel [39], emphasises our findings that donor dependency can limit the flexibility and autonomy of programmes; dominant forces of privilege and oppression shape who is hidden and who is acknowledged; conflict and fragility; political alliances and economic factors influence who can access medicines, where and when; and that the historic legacy of programmes can limit innovation due to the ‘normalisation of processes’. The impact of these factors on effective treatment outcomes for individuals and communities is of course also influenced by individual identities and the intersecting axes of inequity that converge to shape these positions including gender, age, dis/ability, ethnicity, and geography. Health systems are inherently social [40, 41] and gendered [42] thus they become critical mediators in managing the impact that social and structural processes have on individual health outcomes. We identified that health systems bottlenecks, challenges and limitations, sometimes due to lack of consideration and discussion of gender and equity issues have left vulnerable populations underserved in relation to the prevention and treatment of PC NTDs across all types of coverage explored within the Tanahashi framework.

**Availability coverage** can be compromised due to: medicine shortages and delays because of poor requisition estimates and weak supply chain, leading to key populations being missed based on ethnicity (e.g. migrant groups) or religion; policy or programmatic engagement can mean out-of-school children or children in religious schools are missed, showing interlinkages between programme and education structures; and out of pocket payments for frontline health providers can render MDA impoverishing for those delivering drugs within communities. **Accessibility coverage** is hindered by the interconnection of geography, economic factors and unadaptable health systems both in urban and rural settings which can lead to context specific inequities: men were frequently left out of distribution due to condensed distribution periods and absenteeism from communities based on livelihood or economic activities; seasonality of distribution meant that some entire communities, in the most isolated settings, were excluded from distribution, showing an interaction between restrictions to programme adaptability, geography and economic factors; and limited flexibility in distribution strategies can restrict access to interventions for people living with disability, women, and men, as well as those living in urban areas. **Acceptability of medicines** is frequently influenced by: restrictions to autonomous decision making based on gender, age, and religion; socio-economic context (i.e. wealth and poverty) and availability of food can influence the experience of side effects and thus interconnects to shape programme acceptance; and traditional belief systems that are not considered in programme design e.g. the use of sticks for measurement can shape the acceptability of the intervention. **Contact coverage** is frequently impacted by patriarchal decision-making structures which determine who are CDDs as well as who CDDs can interact with. This can result in many women being untreated, or unrecognised by the programme. Finally, **effective coverage** is negatively influenced when pregnant women, people with disabilities, and the chronically sick are frequently left out of programme delivery due to programmatic campaign structures and definitions of ‘ill health’.

Although policy and practice goals e.g. those laid out in the NTD 2020 declaration do not require 100% MDA coverage, instead aiming for between 65% (lymphatic filariasis) and 75% (soil transmitted helminths and schistosomiasis for school aged children) [3], from an equity and ethical perspective this brings challenges. Ultimately, sections of the population are missed and as the combined application of the Tanahashi framework and intersectional analysis shows, it is consistently those experiencing intersecting vulnerabilities that are missed. As an NTD community we need to think beyond the ultimate coverage goal and begin to breakdown the different elements of coverage to understand who is left behind and why. In understanding this, we can begin to think about who it is we need to target to go the ‘extra mile’ to reach those who need it the most and in supporting the remaining steps toward NTD elimination [12, 19, 43, 44]. This would contribute to the progressive realisation of UHC by prioritising the ‘worst off’ [11], thus truly supporting the NTDs communities’ ambitious vision to contribute to UHC attainment and the SDGs. Arguably, it is only when the fundamental right for all to be able to access prevention and treatment for PC NTDs is recognised and realised by the NTD and wider global health community, that PCT NTDs can become a true ‘litmus test’ for UHC.

### So, what does this all mean for programme delivery, integration into routine health systems and UHC?

There are key nuances in equity challenges across contexts, however, our collective analysis emphasises that there are core common issues that require action. There is a key need for multi-pronged strategies to address inequities within NTD programming, ensuring UHC through the equitable attainment of development priorities whilst maximising social inclusion and cohesion [45]. Such strategies should respond to power dynamics at varying levels including at the local, national and global level.

### Local level: Community participation and accountability to challenge power relations

Meaningful community participation strengthens public accountability which is a critical step on the pathway to achieving UHC as it allows for health systems and or health intervention reform to be guided by the needs and values of a broad range of stakeholders, not just the most powerful[11, 46]. The consistent strength of community ideas (embedded within the realities of different contexts) to improve programmes supports this notion and emphasises the original ethos of NTD programming that communities have the knowledge to be able to guide and design the way that programmes are delivered [43]. However, as shown by others[47, 48], in contrast to previous times, communities are now less frequently consulted and thus the effectiveness of interventions can be compromised. We found this to be particularly the case in relation to accessibility and acceptability coverage due to programme weaknesses in the generation of awareness and knowledge about services as well as limited adaptability in the methods for medicine distribution and campaign timing. Rapid levels of urbanisation [49], coupled with increased pockets of fragility and conflict [50] mean that now, more than ever, the flexibility and responsiveness of MDA approaches is essential to reach those who are most in need[20]. We therefore need to begin to think critically about how to re-engage communities in different ways and across a variety of contexts, including the most fragile, to ensure that health systems and interventions are held to account to the needs of the most vulnerable.

Responses must draw on evidence such as that presented here and continue to seek the views and opinions of the most marginalised. Implementation research can provide a space for such evidence informed decision making, and when facilitated effectively can contribute to breaking down power hierarchies between communities and health systems to support in the development of mutual trust and accountability [10]. For example, as shown here, trust in the health system and distributors is critical to improving acceptability coverage, however MDA campaigns tend to sit on the periphery of the health system due to a history of ‘chronic verticalization’[51]. Whilst there is evidence that community leaders were engaged in some contexts, there were still clear gaps in understanding the best way to ensure all community members were engaged, particularly those who experienced marginalisation based on intersecting inequities. Implementation research has the potential to understand accountability structures across contexts, such as health committees and other community-based health units, which can support in decreasing inequities as they frequently engage with vulnerable groups and can support in the provision of community-based supervision. Understanding how to embed MDA campaigns within existing social accountability frameworks will also ensure MDA campaigns that are better integrated within the health system[52].

### National and sub-national level: Supporting intersectional analysis within programmes and enhancing workforce support

At the national and sub-national level, a critical first step to ensure programmatic responsiveness to equity issues is to prioritise the collection of both quantitative and qualitative data. Cohn et al 2019, emphasised that the collection and use of quality quantitative gender disaggregated data is possible, but that accompanying qualitative analysis allows us to obtain a better understanding of ‘whether, how and to what benefit countries have used such data to strengthen programmes’[12]. Health systems must be responsive to changing contexts such as growing urbanisation, changing livelihood patterns, growing fragility and insecurity and unstable political contexts [20, 21]. Space and time within intersectional analysis is a key factor that influences service provision and acceptance and should be built into to annual programme reviews to address change. We need, therefore, to now also consider how gender disaggregated data can be consistently linked to other markers of social inequities such as age, gender, wealth and dis/ability to routinely consider and address the impact of intersecting vulnerabilities within the broader socio-political context. Quantitative data in isolation can mask significant programme inequities or present varying outcomes. WHO’s toolkit ‘Towards universal coverage for preventive chemotherapy for Neglected Tropical Diseases: guidance for assessing “who is left behind and why”?’ is designed to take the initial step toward supporting NTD programme managers to begin to collect and analyse both quantitative and qualitative indicators to support ongoing equity analysis [7], however a question remains regarding the feasibility of this approach for an already overstretched health workforce. New technologies, information systems and innovations are also required to support health system responsiveness [18].

CDDs, as a type of community health worker (CHW), are a critical asset to all NTD programmes in supporting the negotiation of community power dynamics [53, 54], and serve as a key bridge between health systems and the populations to whom they are seeking to provide services. However, for NTD programmes to maximise the benefits that CDDs can provide in negotiating power within communities, policy makers and implementers need to consider gender and equity dynamics in conceptualising the CDD role within MDA. This includes thinking through how to manage the power dynamics associated with CDD selection as well as how gender roles and relations and other equity considerations shape individual experience of being a CDD so that they can be effectively supported [53]. Gender and equity reviews of NTD policies and programmes in specific relation to CDDs would be a critical first step in supporting programmes to adapt to community and individual level power dynamics. Specific training modules or activities could also be integrated within CDD and health worker MDA training processes to support these health cadres to be more responsive to power dynamics at the community level and in encouraging them to become agents of gender and equity transformation [8, 54].

Historical experience of side effects and severe adverse events as described in other studies [19, 43, 44, 47] were a key factor in reducing programme acceptance and presented a multitude of challenges for programme implementers, including teachers. The national and sub-national level must respond to support its workforce to manage these challenges. Consideration of a well-designed system for reporting of side effects and adverse events (including severe), would not only support frontline health workers to respond to these situations but also support in increasing acceptability coverage of interventions. The establishment of clear pharmacovigilance guidelines in Nigeria has been shown to be effective in this regard [48].

### Global level: Redressing power imbalances within the international NTD community

Finally, at the global level, power imbalances between donors, implementing partners and national programmes can limit the autonomy of national actors to be able to respond to needs within their own context. Such limitations to programme adaptability have been shown here to reduce the equity in intervention outcomes, thus the time is now for donors and implementing partners to consider collective action to redress these imbalances. The NTD funding landscape is in a period of transition with the initiation of the DFID funded ‘Accelerating the sustainable control and elimination of NTDs (ASCEND)’ and US AID funded ‘Act to End’ programmes. This together with UHC aspirations to tackle inequality and inequity presents a clear window of opportunity to redress power imbalances amongst the international NTD community. This could be leveraged through adaptations to stringent MDA timings or reporting deadlines; and some flexibility in campaign structure so that specific populations e.g. pregnant women, the chronically ill, and migrant communities can be reached outside of campaign periods, through for example, some medicines being left in health centres to allow for longer term ‘mop up’. Sustaining access to treatment over longer periods for these populations would likely contribute to reduced morbidity burdens associated with these diseases that will remain even after ‘elimination’ when active transmission ceases.

As we move towards the progressive realisation of UHC, these findings should be central to discussions on providing health for all. MDA campaigns have been implemented for many years and the inequities within them are only now being discussed. The lessons from PC-NTD programmes should be heeded by UHC actors who can build in intersectional analysis from the beginning, from selecting services to prioritising how to deliver them. However, for the NTD community to continue to support their vision of being a true ‘litmus test’ for the attainment of UHC, they must continue to seek to understand how to make their programming more equitable across all levels of coverage to ensure that the most vulnerable have continued access to future treatment options.
